# Supplementary material for: Mechanisms governing the pioneering and redistribution capabilities of the non-classical pioneer PU.1
Source: Nat Commun. 2020 Jan 21;11:402. doi: 10.1038/s41467-019-13960-2 (PMC6972792; doi:10.1038/s41467-019-13960-2)
Supplement: Supplementary file 7 — Source data [file 41467_2019_13960_MOESM7_ESM.zip › Source_Data/FigureS3/FigureS3H_MotifScanOutput/homerResults/motif8.similar.html]

motif8

## Information for motif8

A
G
T
C
A
C
G
T
A
C
T
G
A
G
T
C
T
A
C
G
A
C
T
G
A
C
G
T
A
C
G
T
  
Reverse Opposite:  

C
G
T
A
C
G
T
A
A
G
T
C
A
T
G
C
A
C
T
G
G
T
A
C
C
G
T
A
C
T
A
G
  

|  |  |
| --- | --- |
| p-value: | 1e-110 |
| log p-value: | -2.544e+02 |
| Information Content per bp: | 1.882 |
| Number of Target Sequences with motif | 1741.0 |
| Percentage of Target Sequences with motif | 45.03% |
| Number of Background Sequences with motif | 12264.5 |
| Percentage of Background Sequences with motif | 27.38% |
| Average Position of motif in Targets | 297.8 +/- 309.4bp |
| Average Position of motif in Background | 208.9 +/- 142.5bp |
| Strand Bias (log2 ratio + to - strand density) | 0.0 |
| Multiplicity (# of sites on avg that occur together) | 1.28 |
| Motif File: | file (matrix) reverse opposite |

### Similar de novo motifs found

|  |  |  |  |  |  |  |  |
| --- | --- | --- | --- | --- | --- | --- | --- |
| Rank | Match Score | Redundant Motif | P-value | log P-value | % of Targets | % of Background | Motif file |
| 1 | 0.842 | A C G T A C G T A C T G A G T C C T A G A T C G A C G T A C G T C G A T | 1e-57 | -131.272551 | 13.61% | 6.13% | motif file (matrix) |
| 2 | 0.790 | G A T C C T G A G T C A A G T C C G A T A C T G A G T C | 1e-33 | -77.616874 | 48.32% | 38.24% | motif file (matrix) |
| 3 | 0.871 | A G T C A C G T A C T G A G T C A C T G A C T G A C G T | 1e-21 | -50.266295 | 4.22% | 1.68% | motif file (matrix) |
| 4 | 0.839 | T C A G A G T C A C G T A C T G T A G C T A C G A C T G C G A T A G C T C G A T A C T G | 1e-20 | -46.867681 | 4.27% | 1.78% | motif file (matrix) |
